# Geometric Positioning for Satellite Imagery without Ground Control Points by Exploiting Repeated Observation

**DOI:** 10.3390/s17020240

**Published:** 2017-01-26

**Authors:** Zhenling Ma, Xiaoliang Wu, Li Yan, Zhenliang Xu

**Affiliations:** 1 Shanghai Engineering Research Center of Hadal Science and Technology, Research Center for Ocean Mapping and Applications, College of Marine Sciences, Shanghai Ocean University, Shanghai 201306, China; 2 Commonwealth Scientific and Industrial Research Organisation (CSIRO) DATA61, Western Australia 6014, Australia; Xiaoliang.Wu@csiro.au; 3 School of Geodesy and Geomatics, Wuhan University, Wuhan 430079, China; lyan@sgg.whu.edu.cn; 4 China Center for Resources Satellite Data and Application, Beijing 100094, China; xuzhenliang@pku.edu.cn

**Keywords:** geometric positioning, satellite images, repeated observation, new images, without GCPs

## Abstract

With the development of space technology and the performance of remote sensors, high-resolution satellites are continuously launched by countries around the world. Due to high efficiency, large coverage and not being limited by the spatial regulation, satellite imagery becomes one of the important means to acquire geospatial information. This paper explores geometric processing using satellite imagery without ground control points (GCPs). The outcome of spatial triangulation is introduced for geo-positioning as repeated observation. Results from combining block adjustment with non-oriented new images indicate the feasibility of geometric positioning with the repeated observation. GCPs are a must when high accuracy is demanded in conventional block adjustment; the accuracy of direct georeferencing with repeated observation without GCPs is superior to conventional forward intersection and even approximate to conventional block adjustment with GCPs. The conclusion is drawn that taking the existing oriented imagery as repeated observation enhances the effective utilization of previous spatial triangulation achievement, which makes the breakthrough for repeated observation to improve accuracy by increasing the base-height ratio and redundant observation. Georeferencing tests using data from multiple sensors and platforms with the repeated observation will be carried out in the follow-up research.

## 1. Introduction

**Figure 1:**
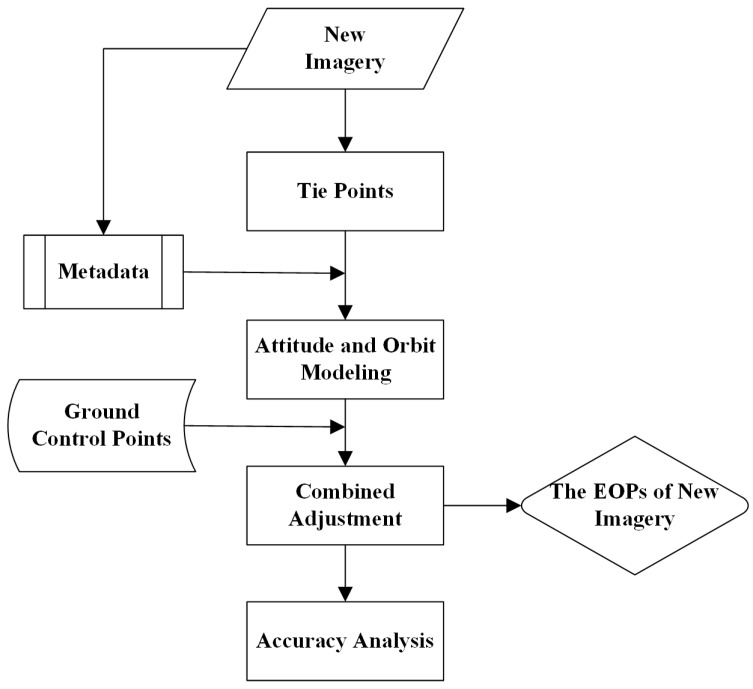
Workflow of the conventional georeferencing method with GCPs.

With the development of space technology and the performance of remote sensors, high-resolution satellites are continuously launched by countries around the world. Due to high efficiency, large coverage and not being limited by the spatial regulation, satellite imagery becomes one of the important means to acquire geospatial information. Geometric processing is the basis of image effective utilization. At present, a high accuracy of georeferencing with satellite imagery is achieved by the conventional block adjustment method with ground control points (GCPs), which is on the basis of the sensor model and the system error model [1,2,3,4], shown in Figure 1. However, GCPs’ acquirement is time consuming and difficult for remote and harsh areas. In recent research, position accuracy for satellite imagery without GCPs has been improved compared with the conventional forward intersection, for example multiple strips of ZY-3 images were tested with bundle block adjustment without GCPs, in which the horizontal and vertical accuracy was about 13–15 m [5]; a hybrid model is used to extract elevation from WorldView stereo data, in which vertical accuracy was about 2.5 m over bare surfaces [6]; Mumtaz proposed a method for the positioning without GCPs considering the thermo-elastic effects on the satellite, which was applied to the UK-DMC images, and a geolocation accuracy of 0.5–1 km was achieved [7]; a combined adjustment method with multiple sources of satellite imagery was presented to improve the accuracy without GCPs [8]. However, they cannot reach the optimal precision, which is comparable to the result of conventional block adjustment with GCPs, and the outcome of existing spatial triangulation is not effectively used in the present methods.

The high accuracy of direct georeferencing for satellite imagery without GCPs can be obtained, either by exterior orientation parameters (EOPs) with high precision, especially pitch, to satisfy the elevation accuracy, which increases the technical difficulty of star sensor design [9,10,11,12], or by an appropriate base-height ratio [13]. Currently, mapping satellites with high resolution around the world observe the Earth repeatedly [14,15,16]. Repeated observation on the same regions leading to a close approximation to the true position is important because it can provide benefits that can increase not only the multi-baseline images from different views, which can reduce the accidental errors, as well as increase redundant observation, but also the base-height ratio.

From these points of view, the Spatial Triangulated Network (STN) is presented [17], which is an extension to the Metric Information Network (MIN) [18,19] and a vessel for the metric information, storing the outcomes of existing spatial triangulation of imagery with a degree of redundancy over an area of interest, including oriented imagery with the EOPs and metadata saved with Extensible Markup Language (XML), as well as 3D coordinates and their error covariance matrix of ground points measured by spatial triangulation, which are stored in matrices. The area covered by the STN can range from a few square miles to the size of countries or continents using imagery from different sensors carried on diverse platforms, such as normal digital camera on the ground, metric cameras on the plane and sensors on the satellites.

The resulting STN can be applied in two ways. First, the stored ground points with a priori error covariance in the STN can be transferred on the new imagery, and then combined adjustment using the linear mean-square estimator is carried out with the new ground points matched from the new imagery, in which the outputs are the 3D coordinates and the a posteriori error covariance of these stored and new ground points. The new ground points are then added in the STN; meanwhile, the existing ones in the STN are updated with the posterior error covariance and new 3D coordinates. Over time, as more new imagery are introduced, not only the number of ground points increases in the area, but the accuracy of these points improves, as well. These points generate the sequential ground control network called MIN in Dolloff’s paper [18,19]. Second, an efficient method of geometric positioning for new stereo imagery without the GCPs is proposed in this paper, using the oriented imagery, which is taken as repeated observation and extracted from the STN. Combined adjustment is executed with the oriented imagery to achieve the EOPs of new non-oriented imagery without GCPs. Then, the new imagery with the EOPs is saved in the STN.

The STN involves several technologies, ranging from the management of the existing spatial triangulation to image retrieval to update of STN to fast processing of the large-scale matrix. This paper derives the positioning model with the oriented imagery, and the experiments prove that the proposed method is superior to the conventional positioning method using SPOT-5 and ZY-3 satellite imagery. Other technologies will be carried out in follow-up research.

## 2. The Mathematical Method

**Figure 2:**
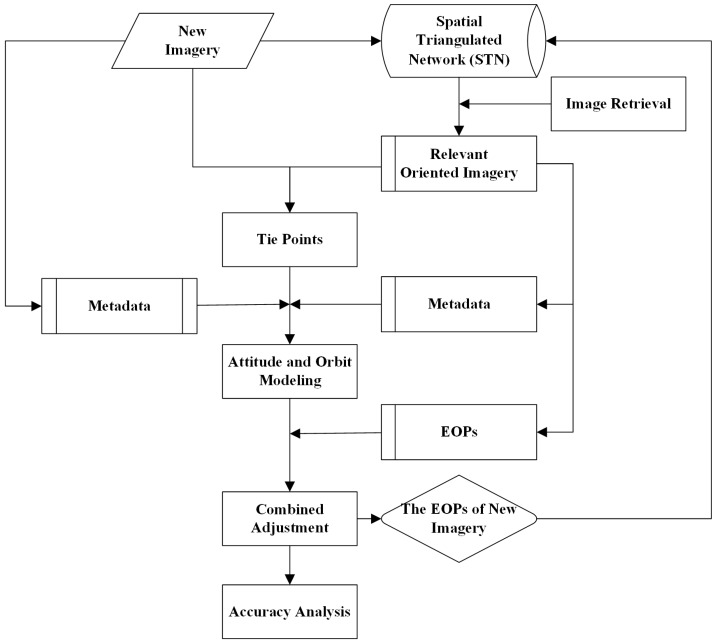
Workflow of georeferencing using the oriented imagery without GCPs.

In this paper, the proposed orientation method for the new imagery using the oriented imagery comprises four parts. First, tie points are acquired by matching new imagery with the oriented imagery. Second, attitude and orbit are modelled based on metadata, and they can be refined with the EOPs of oriented imagery. Third, The EOPs of new imagery and 3D coordinates of the tie points are calculated by combined adjustment. Finally, accuracy is assessed. A flow chart is shown in Figure 2.

### 2.1. The Mathematical Model

Linear array CCD sensors on the satellite for surveying and mapping acquire imagery by push-broom mode, and each line is the result of a perspective projection. The sensor model [20] is shown in Formula (1):

(1) P=S(t)+λ·O(t)·R(t)·M·(p−c)

where ***P*** is the coordinate vector of ground points; *t* is the image line acquisition time; ***S***(*t*) is the vector of satellite positions; *λ* is the scaling factor; **O**(*t*) represents the rotation matrix from the orbit system to the WGS84 system; **R**(*t*) is the rotation matrix from the platform system to the orbit system; **M** is the rotation matrix from the camera system to the platform system; ***p*** represents the coordinate vector of image points; ***c*** is the vector of interior orientation.

The elements of exterior orientation of the linear array CCD sensor are clearly changing at each line; therefore, the image geometry is known as dynamic. Another important feature of a dynamic image is that, although the orientation elements are continually changing, they are changing in a highly predictable way [21]. Investigations, based on simulated orbit data, showed that third order polynomial functions are used for interpolating the orbit and attitude in a short period quite accurately [1], aided by metadata, shown in Formula (2). The interpolated orbit and attitude have deviation with the true values owing to the system errors of the ephemeris, which is compensated by the second polynomial function, shown in Formula (3), in which the refined attitude and orbit at any line are described as the sums of the interpolated values and the system errors:

(2) $$\left\{\begin{array}{c} \;X_{S}^{\mathrm{obs}}\left(\bar{t}\right)=a_{x0}+a_{x1}\cdot \bar{t}+a_{x2}\cdot {\bar{t}}^{2}+a_{x3}\cdot {\bar{t}}^{3}\\ \;Y_{S}^{\mathrm{obs}}\left(\bar{t}\right)=a_{y0}+a_{y1}\cdot \bar{t}+a_{y2}\cdot {\bar{t}}^{2}+a_{y3}\cdot {\bar{t}}^{3}\\ \;Z_{S}^{\mathrm{obs}}\left(\bar{t}\right)=a_{z0}+a_{z1}\cdot \bar{t}+a_{z2}\cdot {\bar{t}}^{2}+a_{z3}\cdot {\bar{t}}^{3}\\ {\mathrm{roll}}^{\mathrm{obs}}\left(\bar{t}\right)=b_{r0}+b_{r1}\cdot \bar{t}+b_{r2}\cdot {\bar{t}}^{2}+b_{r3}\cdot {\bar{t}}^{3}\\ {\mathrm{pitch}}^{\mathrm{obs}}\left(\bar{t}\right)=b_{p0}+b_{p1}\cdot \bar{t}+b_{p2}\cdot {\bar{t}}^{2}+b_{p3}\cdot {\bar{t}}^{3}\\ {\mathrm{yaw}}^{\mathrm{obs}}\left(\bar{t}\right)=b_{y0}+b_{y1}\cdot \bar{t}+b_{y2}\cdot {\bar{t}}^{2}+b_{y3}\cdot {\bar{t}}^{3} \end{array}\right.$$

(3) $$\left\{\begin{array}{c} \;X_{S}\left(\bar{t}\right)=X_{S}^{\mathrm{obs}}\left(\bar{t}\right)+c_{x0}+c_{x1}\cdot \bar{t}+c_{x2}\cdot {\bar{t}}^{2}\\ \;Y_{S}\left(\bar{t}\right)=Y_{S}^{\mathrm{obs}}\left(\bar{t}\right)+c_{y0}+c_{y1}\cdot \bar{t}+c_{y2}\cdot {\bar{t}}^{2}\\ Z_{S}\left(\bar{t}\right)=Z_{S}^{\mathrm{obs}}\left(\bar{t}\right)+c_{z0}+c_{z1}\cdot \bar{t}+c_{z2}\cdot {\bar{t}}^{2}\\ \mathrm{roll}\left(\bar{t}\right)={\mathrm{roll}}^{\mathrm{obs}}\left(\bar{t}\right)+e_{r0}+e_{r1}\cdot \bar{t}+e_{r2}\cdot {\bar{t}}^{2}\\ \;\mathrm{pitch}\left(\bar{t}\right)\;\;={\mathrm{pitch}}^{\mathrm{obs}}\left(\bar{t}\right)+e_{p0}+e_{p1}\cdot \bar{t}+e_{p2}\cdot {\bar{t}}^{2}\\ \;\mathrm{yaw}\left(\bar{t}\right)={\mathrm{yaw}}^{\mathrm{obs}}\left(\bar{t}\right)+e_{y0}+e_{y1}\cdot \bar{t}+e_{y2}\cdot {\bar{t}}^{2} \end{array}\right.$$

where $X_{S}^{\mathrm{obs}}$, $Y_{S}^{\mathrm{obs}}$, $Z_{S}^{\mathrm{obs}}$, ${\mathrm{roll}}^{\mathrm{obs}}$, ${\mathrm{pitch}}^{\mathrm{obs}}$, ${\mathrm{raw}}^{\mathrm{obs}}$ represent the interpolated orbit and attitude; ($a_{x0}$, $a_{x1}$,..., $b_{y3}$) refers to the coefficients of third order polynomial; $X_{S}$, $Y_{S}$, $Z_{S}$, *roll*, *pitch*, *yaw* represent the refined orbit and attitude; ($c_{x0}$, $c_{x1}$,..., $e_{y3}$) are the coefficients of system error model, treated as EOPs in this paper and known for the oriented imagery, but unknown for the new imagery, shown in Figure 2; $\bar{t}$ is described as Formula (4), in which *t* is image line acquisition time; $t^{0}$ and $t^{E}$ represent the time extremes of the image.

(4) $$\bar{t}=\frac{t-t^{0}}{t^{E}-t^{0}}$$

The observation equations is obtained when Formula (3) is substituted into Formula (1) and linearized using the first order of the Taylor series expansion, shown in Formula (5), which is the observation equation of the conventional block adjustment:

(5) V=At+Bx−lP

where *V* refers to the residual vector; **A** is the coefficient matrix for EOPs; **B** is the coefficient matrix for tie points; *x*, *t* are vectors of unknown corrections for tie points and EOPs; vector *l* represents the difference between the observed image point and the calculated value; **P** is the weight for image point observation.

The observation equations of the georeferencing method using oriented imagery in this paper are obtained according to Formula (5), shown in Formulas (6) and (7). It represents the observation equation of tie points on the oriented imagery in Formula (6), in which EOPs are known and only coordinates of tie points are unknown. The observation equation of tie points on the new imagery is shown in Formula (7), in which the EOPs and coordinates are the unknowns:

(6) $$V_{T}=B_{T}x\;\;-l_{T}\;P_{T}$$

(7) $$V_{N}=A_{N}t+B_{N}x-l_{N}\;P_{N}$$

where $V_{T}$, $V_{N}$ are residual vectors for tie points on the oriented and new imagery, respectively; $B_{T}$, $B_{N}$ are coefficient matrices for the corrections of tie points; $A_{N}$ is the coefficient matrix for EOPs corrections of the new imagery; vector $l_{T}$ is the difference between the observed image points and the calculated value on the oriented imagery; vector $l_{N}$ is the difference between the observed image points and the calculated value on the new imagery; weights $P_{T}$, $P_{N}$ are standing for image points on the oriented and new imagery. To avoid instability caused by correlation among the EOPs due to the high flight height and narrow viewing angle of satellite linear array sensors leading to multicollinearity [22], EOPs are introduced into observation equations as pseudo-observations [23], shown in Formula (8):

(8) $$V_{t}=E_{t}t-l_{t}\;P_{t}$$

where $V_{t}$ is the residual vector for EOPs; $E_{t}$ is the coefficient matrix for EOPs’ corrections, which is a unit matrix; vector $l_{t}$ is the difference between the observed EOPs and the calculated value; $P_{t}$ is the weight for EOPs.

The simultaneous observation equation of Formulas (6)–(8) with the matrix of weight is shown in Formula (9):

(9) $$\left[\begin{array}{c} V_{T}\\ V_{N}\\ V_{t} \end{array}\right]=\left[\begin{array}{cc} 0 & B_{T}\\ A_{N} & B_{N}\\ E_{t} & 0 \end{array}\right]\left[\begin{array}{c} t\\ x \end{array}\right]-\left[\begin{array}{c} l_{T}\\ l_{N}\\ l_{t} \end{array}\right]\;\left[\begin{array}{ccc} P_{T} & & \\ & P_{N} & \\ & & P_{t} \end{array}\right]$$

### 2.2. Combined Block Adjustment

The STN contains a series of overlapped oriented imagery eventually covering the region of interest. The image retrieval method is used to acquire the oriented imagery from the STN overlapped with the new imagery. The image extents are extracted from the metadata firstly, which is determined by the four vertexes position described as geographic coordinates in the World Geodetic System 1984 (WGS 84). Secondly, intersections are detected between the oriented and new images on the basis of the vertexes coordinates. Thirdly, the intersecting oriented images are introduced into the combined block adjustment. Tie points, identified on the oriented and new imagery, are necessary for the combined block adjustment. Least squares matching (LSM) techniques are used to automatically obtain the corresponding feature points. The main steps of LSM are: (1) the matching using the pyramid structure with the correlation coefficient is executed firstly, in which the corresponding points are searched within a square window of 5 × 5 pixels, and the size is related to the magnitude of parallax; the correlation size is set to 8 × 8 pixels; (2) least square matching techniques are applied to ensure that the quality of the corresponding matched points is accurate to approximately 0.1–0.2 pixels. The window size is set to 5 × 5 in pixels for least square matching. The coefficient limit is set to 0.8 used to determine whether or not two points are to be considered as possible matches. At last, 133 unique tie points are found throughout the overlapping area of the imagery.

In the simultaneous observation equation of the geopositioning method of Equation (9), the weights $P_{T}$, $P_{N}$, $P_{t}$ represent the contribution of the observation in the adjustment process. Weights $P_{T}$, $P_{N}$ are determined by the measurement accuracy of corresponding image points, which are related to the precision of LSM approximate to 0.1 pixels for the datasets in this paper and expressed as the pixel size, namely, image resolution. The weight of the image point with the highest resolution is set to one. Others are on the basis of highest resolution-to-resolution ratio. The weight $P_{t}$ is determined by the ratio between the variance of point’s observation and orientation parameters, in which the variance of orientation parameters is determined by the measurement precision of attitude and orbit. In this paper, EOPs are introduced into the observation equations as pseudo-observations [23] to improve the rank defect of normal equation, which is derived from Formula (9), shown in Formula (10):

(10) $$\left[\begin{array}{cc} N_{11} & N_{12}\\ N_{21} & N_{22} \end{array}\right]\left[\begin{array}{c} t\\ x \end{array}\right]\;=\;\;\;\left[\begin{array}{c} A_{N}^{T}P_{N}l_{N}+P_{t}l_{t}\\ B_{T}^{T}P_{T}l_{T}+B_{N}^{T}P_{N}l_{N} \end{array}\right]$$

where:

(11) $$N_{11}=A_{N}^{T}P_{N}A_{N}+P_{t},\;\;\;\;N_{12}=A_{N}^{T}P_{N}B_{N},\;\;\;\;N_{21}=B_{N}^{T}P_{N}A_{N},\;\;\;\;N_{22}=B_{T}^{T}P_{T}B_{T}+B_{N}^{T}P_{N}B_{N}$$

The weight $P_{t}$ is added in $N_{11}$ after the introduction of pseudo-observations, which can improve the state of normal equation and ensures the stability of the solution.

The combined block adjustment includes two steps. First, the initial value of tie points and EOPs are determined, in which the coordinates of tie points are calculated with forward intersection using the interpolated orbit and attitude, and the initial EOPs standing for the coefficients of system error model (Formula (3)) are set to 0. The vectors of unknown corrections *x*,*t* are solved from the Formula (10) with the least square estimation, shown in Formula (12):

(12) $$\left[\begin{array}{c} t\\ x \end{array}\right]\;={\left[\begin{array}{cc} N_{11} & N_{12}\\ N_{21} & N_{22} \end{array}\right]}^{-1}\;\left[\begin{array}{c} A_{N}^{T}P_{N}l_{N}+P_{t}l_{t}\\ B_{T}^{T}P_{T}l_{T}+B_{N}^{T}P_{N}l_{N} \end{array}\right]$$

Some GCPs are taken as check points, with which the accuracy is assessed. The calculated coordinates of these points can be achieved with the EOPs after block adjustment. Accuracy assessment is performed by root mean squared error (RMSE) according to the difference of truth and calculated coordinates of the check points, shown in Formula (13):

(13) $$\left\{\begin{array}{c} \mu_{X}=\sqrt{\frac{\sum {\left(X_{g}-X_{c}\right)}^{2}}{n}}\\ \mu_{Y}=\sqrt{\frac{\sum {\left(Y_{g}-Y_{c}\right)}^{2}}{n}}\\ \mu_{Z}=\sqrt{\frac{\sum {\left(Z_{g}-Z_{c}\right)}^{2}}{n}} \end{array}\right.$$

where $\mu_{X}$, $\mu_{Y}$, $\mu_{Z}$ refer to the RMSE of check points with three directions; *n* refers to the number of check points; $X_{g}$, $Y_{g}$, $Z_{g}$ are the actual ground coordinates of check points; $X_{c}$, $Y_{c}$, $Z_{c}$ are the calculated coordinates of check points.

## 3. Experimental Results and Analysis

**Figure 3:**
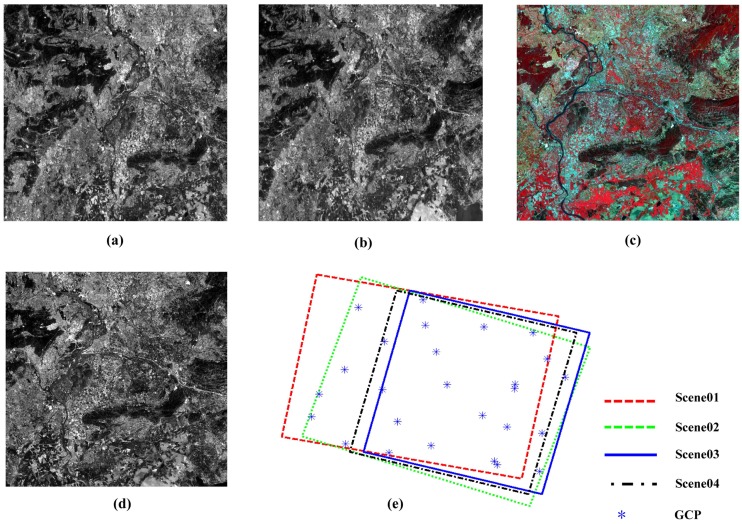
Four SPOT-5 images and the overlapping area : (**a**) Scene 01; (**b**) Scene 02; (**c**) Scene 03; (**d**) Scene 04; (**e**) The extents of four images and the distribution of GCPs.

**Figure 4:**
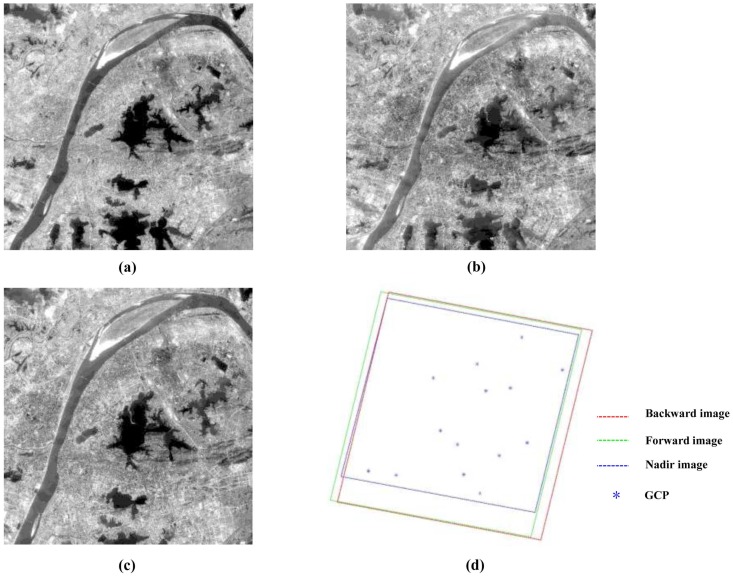
Three ZY-3 images and the distribution of ground truth points: (**a**) Backward; (**b**) Forward; (**c**) Nadir; (**d**) The extents of three images and the distribution of GCPs.

In this study, two groups of datasets are taken as experimental data. The first group contains four SPOT-5 images with different time and spatial resolution taken as experimental data covering an area of France, including two images acquired from the High Resolution Stereoscopic sensor (HRS) with the resolution of 5 m × 10 m and an image obtained from the High Resolution Geometric sensor (HRG) with the resolution of 10 m and an image with the resolution of 2.5 m gained by the super-resolution image processing technique. Twenty six ground truth points (GPS surveyed) are available for GCPs and accuracy assessment, shown in Figure 3. It shows the four images and the overlapping area between them in Figure 3. The second group includes three ZY-3 images covering an area of China, containing two images acquired from the backward and forward TDI CCD sensors with the resolution of 3.6 m and the viewing angle of 22${}^{\circ }$ and an image gained from the nadir TDI CCD sensor with the resolution of 2.1 m. Fourteen ground points measured via GPS are used for GCPs and accuracy assessment, shown in Figure 4. Information about the images is shown in Table 1 and Table 2.

Three experiments are carried out to verify the proposed method in this paper. The first experiment tests new images with conventional adjustment method, and it consists of six cases with SPOT-5 images and four cases with ZY-3 images, where images have different resolutions and base-height ratios. The second one aims at direct georeferencing for stereo pair with the oriented images containing two cases with three subcases for SPOT-5 imagery and three cases for ZY-3 imagery, in which the result is compared with the first test. The third test is positioning for a single image with the oriented images, including two cases with two subcases for SPOT-5 imagery and three cases for ZY-3 imagery. In every subcase, different oriented images are integrated. Images for adjustment are given in Table 1, Table 2, Table 3, Table 4, Table 5, Table 6, Table 7 and Table 8.

### 3.1. Conventional Image Positioning Method

#### 3.1.1. Conventional Block Adjustment with SPOT-5

**Figure 5:**
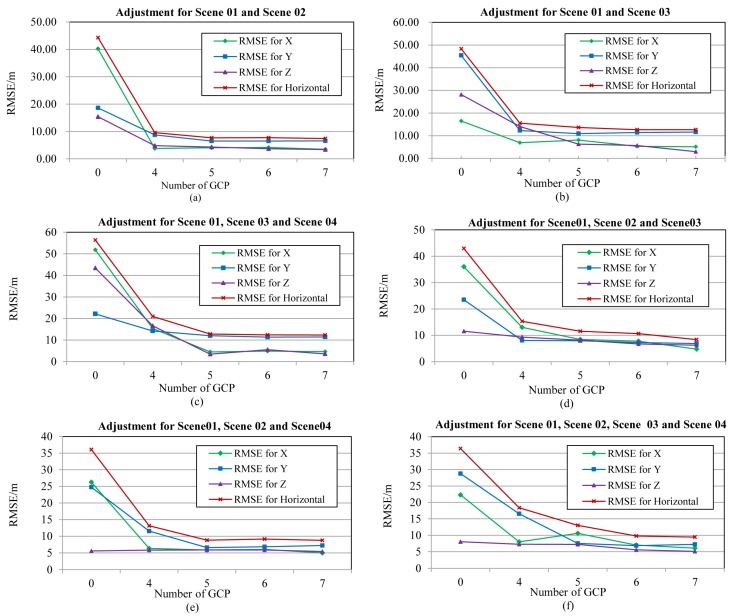
Result of conventional image positioning with different numbers of GCPs using SPOT-5 images: (**a**) accuracy of adjustment for Scenes 01 and 02 with different number of GCPs; (**b**) accuracy of adjustment for Scenes 01 and 03 with different number of GCPs; (**c**) accuracy of adjustment for Scenes 01, 03 and 04 with different number of GCPs; (**d**) accuracy of adjustment for Scenes 01, 02 and 03 with different number of GCPs; (**e**) accuracy of adjustment for Scenes 01, 02 and 04 with different number of GCPs; (**f**) accuracy of adjustment for Scenes 01–04 with different number of GCPs.

Conventional block adjustment is executed for SPOT-5 and ZY-3 imagery. The dataset and positioning accuracy with SPOT-5 are shown in Table 3 and Figure 5.

Forward intersection with interpolated and non-refined orbit and attitude is performed for each case without GCP to gain the position of tie points, shown in Figure 5. Horizontal errors were more than 36 m, and vertical errors were more than 5 m for all of the cases, which were greater than five pixels, mainly caused by the system errors of the orbit and attitude. Case 5 provided the best results, in which the horizontal and vertical accuracy could achieve 36.10 m and 5.57 m, respectively, shown in Figure 5e, while there is a sharp decrease in accuracy in Cases 2 and 3, shown in Figure 5b–c, where their horizontal accuracy reached 48.33 m and 56.38 m, vertical accuracy reaching 28.15 m and 43.39 m because of dataset in Case 5 having a higher base-height ratio with 1.2 and better point measured accuracy due to the higher spatial resolution. The accuracy of other cases was between these three cases. Horizontal accuracy was 36.41 m, 42.97 m and 44.33 m, while vertical accuracy was 8.05 m, 11.53 m and 15.41 m for Case 6, Case 4 and Case 1, respectively, shown in Figure 5f,d,a.

Block adjustment is carried out with system errors compensated by Formula (3), and different numbers of GCP from 4–7 are applied to the adjustment, shown in Figure 5. Accuracy improved greatly when the number of GCP was less than five and maintained stable when the quantity was more than five. Horizontal and vertical accuracy could increase to about 10 m and within 6 m, respectively, for all of the cases.

#### 3.1.2. Conventional Block Adjustment with ZY-3

**Figure 6:**
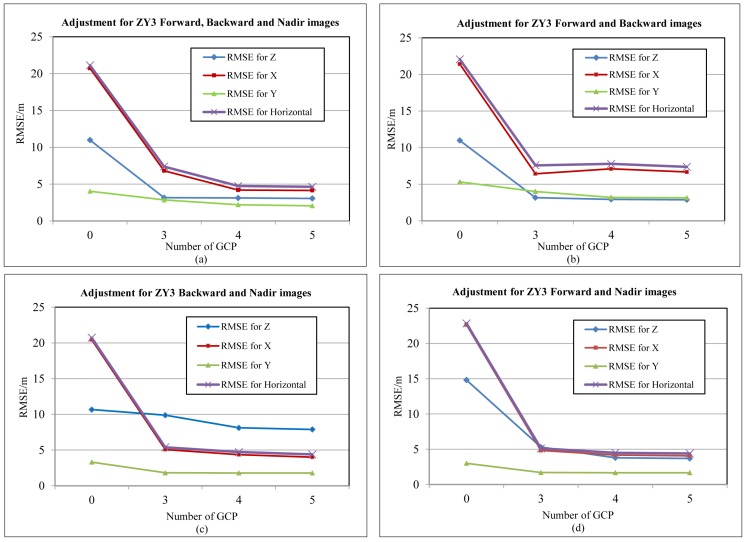
Result of conventional image positioning with different numbers of GCPs using ZY-3 images: (**a**) accuracy of adjustment for forward, nadir and backward images with different number of GCPs; (**b**) accuracy of adjustment for backward and forward images with different number of GCPs; (**c**) accuracy of adjustment for backward and nadir images with different number of GCPs; (**d**) accuracy of adjustment for forward and nadir images with different number of GCPs.

Another dataset of ZY-3 images (Table 4) is also used for the traditional block adjustment, and the positioning accuracy is shown in Figure 6.

Forward intersection is carried out to achieve the georeferencing accuracy without GCPs, and the result is shown in Figure 6. Horizontal and vertical accuracy were more than 20 m and 10 m for all of the cases, which were greater than three pixels, mainly caused by the system errors of the orbit and attitude ubiquitous in the satellite system. Block adjustment is executed, and different numbers of GCP from 3–5 are applied to the adjustment, shown in Figure 6. Accuracy improved greatly when using GCP to compensate the system error. Horizontal and vertical accuracy could increase to about 5 m and 8 m, respectively, for all of the cases.

### 3.2. Direct Georeferencing for Stereo Pairs with the Oriented Imagery

The second test is direct georeferencing for new stereo image pairs with the oriented images treated as repeated observation, whose accuracy is compared with the conventional method. The test contains two examples conducted with SPOT-5 and ZY-3 satellite images, respectively.

#### 3.2.1. The Cases with SPOT-5 Images

**Figure 7:**
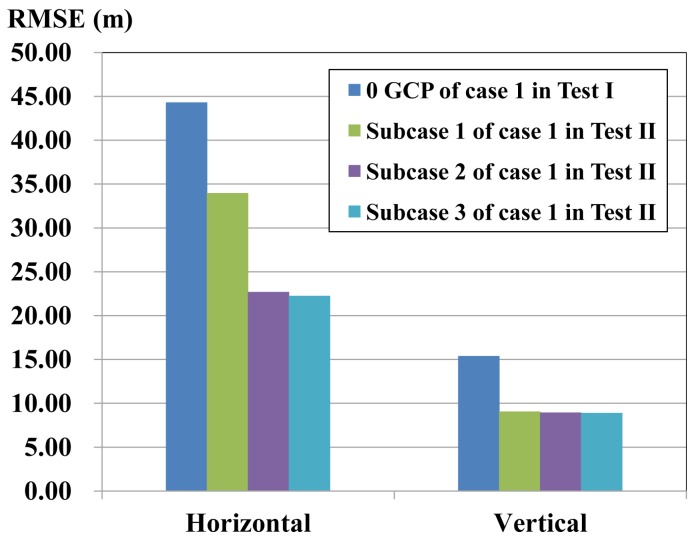
Accuracy Comparison between Case 1 of Test II and Test I.

**Figure 8:**
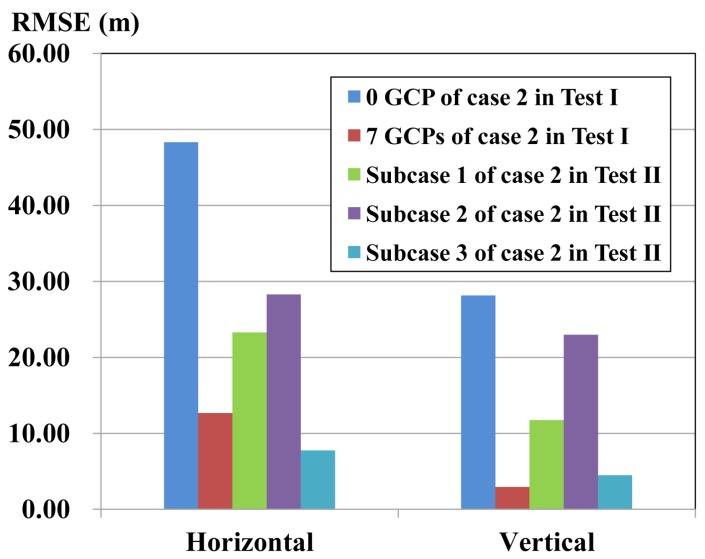
Accuracy comparison between Case 2 of Test II and Test I.

The first test example contains two cases with three subcases for SPOT-5 imagery, shown in Table 5. The accuracy comparison is shown in Figure 7 and Figure 8.

In Case 1, the new stereo image pair is constituted with Scenes 01 and 02 having the highest base-height ratio among the four images, and different oriented images, Scene 03, Scene 04 and both Scene 03 and Scene 04, are integrated into the adjustment, respectively, which are assigned as Subcases 1, 2 and 3 in Test II in Figure 7 and Table 5.

These three subcases perform better than those of conventional forward intersection of Scenes 01 and 02 shown in Case 1 of Test I, in which the horizontal accuracy improvement is 10.44 m, 21.62 m and 22.26 m, respectively, and the vertical accuracy enhancement is about 6 m for all of the subcases, shown in Figure 7. That is because oriented scenes are integrated into adjustment with the new stereo pair, which increases the redundant observation and is helpful for the improvement of accuracy. However, they cannot reach the accuracy of conventional block adjustment with GCPs of Case 1 in Test I (Figure 5a).

Subcases 2 and 3 perform best among the three subcases, in which the horizontal and vertical accuracy was about 22 m and 9 m, respectively, 11 m better than Subcase 1 for the horizontal accuracy, shown in Figure 5, because oriented Scene 04 has a higher spatial resolution than Scene 03, resulting in a better point measured precision. They have approximate vertical accuracy due to the same base-height ratio.

The horizontal accuracy has also an improvement of above 10 m for these three subcases compared with Cases 4, 5 and 6 in Test I (Figure 5d–f), because oriented images have accurate orientation parameters, lines of sight from which are close to the true position. Intersection of all of the lines of sight could be approximate to the true location while combining adjustment.

In Case 2, there are also three subcases. The new image pair is composed of Scenes 01 and 03. Scene 02, Scene 04 and both Scene 02 and Scene 04 with refined orientation parameters is combined for georeferencing with the new pair, assigned as Subcases 1, 2 and 3, respectively, in Figure 8 and Table 5. These three subcases have also better accuracy than the conventional forward intersection of Scenes 01 and 03 shown in Case 2 of test I (Figure 8), due to the increase of redundant observation and base-height ratio. The improvement of horizontal accuracy is 25.04 m, 20.02 m and 40.56 m, respectively, and 16.41 m, 5.17 m and 23.66 m for vertical accuracy. Subcase 3 achieves 5 m better horizontal accuracy than conventional block adjustment with seven GCPs shown in Case 2 of Test I, shown in Figure 8.

The accuracy for Subcase 1 is better than Subcase 2, because the base-height ratio increases from 0.6–1.2 when Scene 02 is involved. The accuracy for Subcase 3 is enhanced greatly, in which horizontal and vertical accuracy reaches 7.77 m and 4.49 m, respectively, owing to the increase of the base-height ratio and the redundant observation. The accuracy can be further improved while more oriented images with higher spatial resolution from different view directions are integrated for positioning.

Subcase 1 doubled its horizontal accuracy compared with the conventional forward intersection of three non-oriented images of Scenes 01, 02 and 03 shown in Case 4 of Test I (Figure 5d), because oriented Scene 02 with accuracy orientation parameters can improve the intersection accuracy than non-oriented Scene 02. Therefore, Subcases 2 and 3 can also achieve better accuracy than the conventional forward intersection of non-oriented images.

#### 3.2.2. The Cases with ZY-3 Images

**Figure 9:**
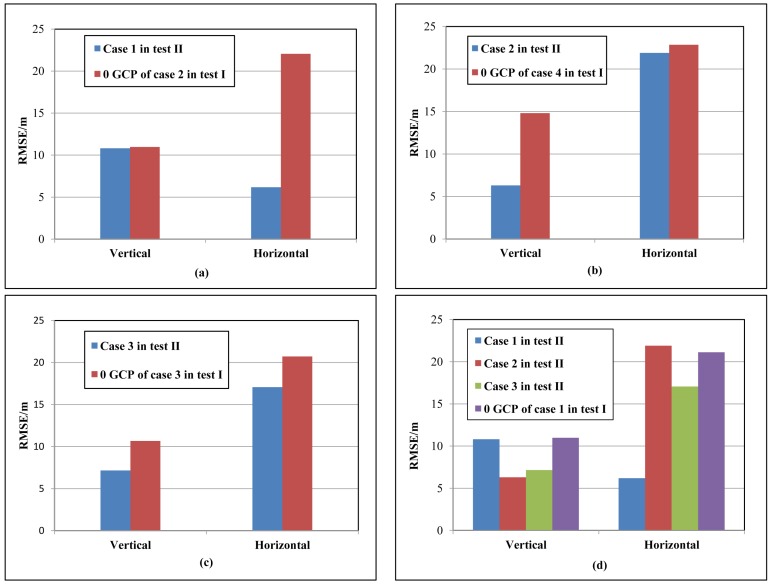
Accuracy Comparison between Case 2 of Test II and Test I with ZY-3 images: (**a**) accuracy comparison of Case 1 in Test II with Case 2 in Test I without GCPs; (**b**) accuracy comparison of Case 2 in Test II with Case 4 in Test I without GCPs; (**c**) accuracy comparison of Case 3 in Test II with Case 3 in Test I without GCPs (**d**) accuracy comparison of three cases in Test II with Case 1 in Test I without GCPs.

The second test example includes three cases with ZY-3 imagery, shown in Table 6. The accuracy comparison is shown in Figure 9.

In Case 1, the new stereo image pair is constituted with backward and forward images of ZY-3 having the highest base-height ratio among the three images. Combined block adjustment is carried out with the new pairs and the oriented nadir image. The horizontal accuracy has an improvement of 15.88 m while the vertical accuracy having an approximate value compared with forward intersection without GCPs of Case 2 in Test I (Figure 6b), shown in Figure 9a. That is because the oriented nadir scene is integrated into adjustment with the new stereo pair, which increases the redundant observation and is helpful in the improvement of accuracy. There is no change of the base-height ratio, so the vertical accuracy remains stable.

In Case 2, the oriented backward image is introduced into the adjustment with the new pairs of forward and nadir images. The vertical accuracy reaches 6 m, 8.51 m better than Case 4 of Test I (Figure 6d) without GCPs owing to the increase of base-height ratio, shown in Figure 9b. The horizontal accuracy has a small improvement of 0.95 m due to the increase of redundant observation.

In Case 3, the new image pair is composed of the backward and nadir image. The horizontal and vertical accuracy improvement was 3.67 m and 3.51 m compared with the forward intersection of Case 3 in Test I (Figure 6c), which is obtained from the combined adjustment with the oriented forward image leading to the increase of the base-height ratio and redundant observation, shown in Figure 9c.

These three cases perform better than the conventional forward intersection of backward, forward and nadir images, shown in Figure 9d, in which the vertical accuracy improvement is 0.16 m, 4.67 m and 3.82 m, respectively, and the horizontal accuracy enhancement is 14.94 m and 4.07 m for Case 1 and Case 3; meanwhile, the horizontal accuracy remains the same for Case 2. Because the oriented image has accurate orientation parameters, the lines of sight from it are close to the true position and benefit the accuracy improvement of combined adjustment.

### 3.3. Direct Georeferencing for a Single Image with the Repeated Observation

The third test is direct georeferencing for the new single image with the oriented images, including two examples with SPOT-5 and ZY-3 images.

#### 3.3.1. The Example with SPOT-5 Images

**Figure 10:**
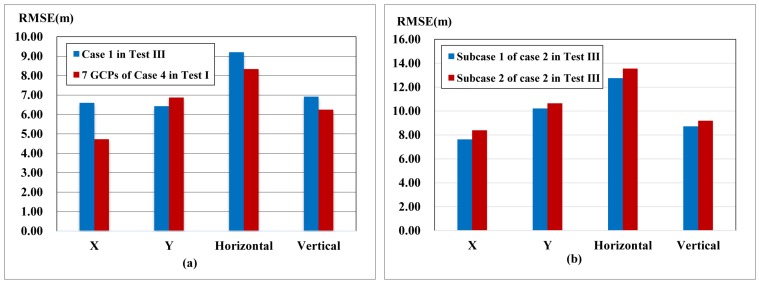
Accuracy of Test III: (**a**) accuracy comparison between Case 1 in Test III and Case 4 in Test I with seven GCPs; (**b**) accuracy comparison between Subcase 1 and Subcase 2 of Case 2 in Test III.

In Case 1, new Scene 03 is oriented with two oriented images of Scenes 01 and 02 and then introduced into Subcase 1 of Case 2 (see Table 7). Scene 03 for Subcase 2 of Case 2 is achieved by conventional adjustment of Test I with seven GCPs. The result of Test III is shown in Figure 10.

The horizontal and vertical accuracy was 9.21 m and 6.92 m in Case 1, approximate to the conventional method with seven GCPs shown in Case 4 of Test I (Figure 10a), because two oriented images played key roles in the intersection accuracy. In Case 2, the horizontal and vertical accuracy of Subcase 2 decreased by only 0.8 m and 0.47 m compared with Subcase 1, shown in Figure 10b, illustrating the oriented Scene 03 from Case 1 has close accuracy with the conventional adjustment method. Thus, a single image can be positioned with the oriented images, which provides another positioning method for a single image.

#### 3.3.2. The Example with ZY-3 Images

**Figure 11:**
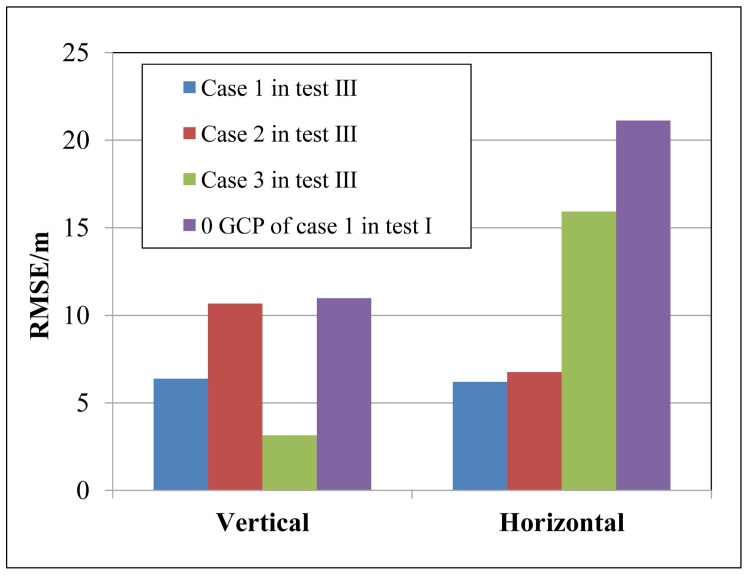
Accuracy comparison between Test III and Case 1 of Test I.

The second test example contains three cases with ZY-3 images, in which the block adjustments are carried out with two oriented images and a new image, shown in Table 8. The accuracy is compared with the conventional block adjustment of these three images, shown in Figure 11.

The accuracy of these three cases is better than the forward intersection in Case 1 of Test I, in which the horizontal accuracy enhancement reaches 14.93 m, 14.37 m and 5.19 m and the vertical accuracy improvement is 4.59 m, 0.30 m and 7.83 m for Case 1, Case 2 and Case 3, respectively, shown in Figure 11. The vertical accuracy of Case 3 performs best among the three cases and is close to the Case 1 of Test I with GCPs owing to the two oriented images with the precise EOPs have the highest base-height ratio. The horizontal accuracy of Cases 1 and 2 is approximate to Case 1 of Test I with three GCPs, due to the backward image with the optimal resolution consisting of the two oriented images.

## 4. Discussion and Conclusions

In this paper, according to the repeated observation of satellites, we have presented that the outcome of spatial triangulation of images is introduced into georeferencing without GCPs as repeated observation, which can guarantee the effective utilization of previous achievement. We have deduced the model for direct georeferencing with the repeated observation. As shown by the experimental results, direct georeferencing for stereo images or a single image using oriented images without GCPs has the advantage over the conventional forward intersection in location accuracy, and it can achieve the approximation to the accuracy of conventional block adjustment with GCPs, which extends previous research on accuracy improvement. To date, since little research has been conducted on repeated observation for improvement of accuracy by increasing the base-height ratio and redundant observation, the spatial triangulated network (STN) can be established, managing the outcome of the spatial triangulation of images. Combined adjustment with the STN and new non-oriented images is conducted to achieve EOPs of new non-oriented images and 3D coordinates of tie points without GCPs. STN will then be updated with the new oriented images. The method using STN cannot only provide the solution to georeferencing without GCPs, but also improve the effective utilization of the spatial triangulation outcome. STN based on repeated observation around the world can provide a way to global topographic mapping without GCPs.

As the goal of this research was exploratory, there existed a limitation: single data from SPOT-5. However, each mapping satellite revolves in its own unique way, thus leading to different resolution and accuracy of positioning. It is recommended that there is a need for multiple sensor data for georeferencing tests with the repeated observation. The establishment, retrieval and update of STN with multi-sensor data should be further approached. According to the results in this paper, the positioning accuracy with repeated observation can attain the close precision with GCPs owing to the increase of base-height ratio and redundant observation, but not all of the tests can gain such results; therefore, it is necessary to do some follow-up studies on determining the scale of STN, in which the highest base-height ratio and redundant observation can be obtained for the purpose of the optimal accuracy. 

## Figures and Tables

**Table 1:** Information about SPOT-5 used in the experiment.

SPOT-5 Imagery	Scene 01	Scene 02	Scene 03	Scene 04
Acquisition date	15 August 2002	18 August 2002	14 August 2004	19 July 2002
Viewing angle (${}^{\circ }$)	26.65	26.13	1.72	1.85
Resolution (m)	5 × 10	5 × 10	10	2.5
Image size (pixels)	12,000 × 12,000	12,000 × 12,000	6000 × 6000	12,000 × 12,000

**Table 2:** Information about ZY-3 used in the experiment.

ZY-3 Imagery	Backward	Forward	Nadir
Acquisition date	16 February 2013	16 February 2013	16 February 2013
Resolution (m)	3.6	3.6	2.1
Image size (pixels)	16,306 × 16,384	16,306 × 16,384	24,516 × 24,576

**Table 3:** SPOT-5 images for georeferencing with the conventional method.

Test	Case	New Imagery
I	1	Scene 01, Scene 02
	2	Scene 01, Scene 03
	3	Scene 01, Scene 03, Scene 04
	4	Scene 01, Scene 02, Scene 03
	5	Scene 01, Scene 02, Scene 04
	6	Scene 01, Scene 02, Scene 03, Scene 04

**Table 4:** ZY-3 images for georeferencing with the conventional method.

Test	Case	New Imagery
I	1	Backward, forward, nadir images
	2	Backward and forward images
	3	Backward and nadir images
	4	Forward and nadir images

**Table 5:** SPOT-5 datasets for direct georeferencing with the oriented imagery.

Test	Case	Subcase	New Imagery	Oriented Imagery
II		1	Scene 01, Scene 02	Scene 03
	1	2	Scene 01, Scene 02	Scene 04
		3	Scene 01, Scene 02	Scene 03, Scene 04
		1	Scene 01, Scene 03	Scene 02
	2	2	Scene 01, Scene 03	Scene 04
		3	Scene 01, Scene 03	Scene 02, Scene 04

**Table 6:** ZY-3 datasets for direct georeferencing with the oriented imagery.

Test	Case	New Imagery	Oriented Imagery
	1	Backward and forward images	Nadir image
II	2	Forward and nadir images	Backward image
	3	Backward and nadir images	Forward image

**Table 7:** SPOT-5 datasets for direct georeferencing with the oriented imagery.

Test	Case	Subcase	New Imagery	Oriented Imagery
III	1	-	Scene 03	Scene 01, Scene 02
	2	1	Scene 04	Scene 01, Scene 02, Scene 03 (from Case 1)
		2	Scene 04	Scene 01, Scene 02, Scene 03 (from conventional adjustment)

**Table 8:** ZY-3 datasets for direct georeferencing with the oriented imagery.

Test	Case	New Imagery	Oriented Imagery
III	1	Forward image	Backward and nadir images
	2	Backward image	Forward and nadir images
	3	Nadir image	Forward and backward images
